# Study of the Effect of Titanium Dioxide Hydrosol on the Photocatalytic and Mechanical Properties of Paper Sheets

**DOI:** 10.3390/ma13061326

**Published:** 2020-03-14

**Authors:** Roberta Grazia Toro, Mohamed Diab, Tilde de Caro, Mona Al-Shemy, Abeer Adel, Daniela Caschera

**Affiliations:** 1 CNR-ISMN, Area della Ricerca Roma 1, 00015 Monterotondo, Italy; tilde.decaro@cnr.it (T.d.C.); daniela.caschera@cnr.it (D.C.); 2 Cellulose and Paper Department, National Research Center, Giza 12622, Egypt; diabism@yahoo.com (M.D.); mona.alshemy@yahoo.com (M.A.-S.); abeermadel2003@yahoo.com (A.A.)

**Keywords:** TiO_2_ hydrosol, photocatalytic paper, sol–gel process, mechanical properties

## Abstract

Different amounts of a stable aqueous TiO_2_ hydrosol were used to fabricate paper sheets having photocatalytic activity. The TiO_2_ hydrosol was prepared in aqueous medium using titanium butoxide as precursor and acetic acid as catalyst for the hydrolysis of titanium butoxide. An aging process at room temperature and atmospheric pressure was finally applied to obtain crystalline anatase TiO_2_ hydrosol. The effects of different TiO_2_ hydrosol loadings on the mechanical strength and barrier properties of modified paper sheets were investigated in detail. The photocatalytic behavior of TiO_2_-modified paper sheets was investigated as well using methylene blue (MB) as target pollutant.

## 1. Introduction

The increase in environmental pollution related in part to the rapid growth of population as well as industrialization poses a serious concern for human life because of its negative effects on natural elements that are vital for life itself such as air and water. In this scenario, photocatalysis can be considered a valid solution to mitigate pollution and to give a sustainable alternative for environmental concerns [1].

TiO_2_ has been widely investigated as promising photocatalyst because of its relatively low cost, high photocatalytic activity, excellent optical properties, high physical and chemical stability, non-corrosive, lack of toxicity, and high availability [2,3,4,5,6,7]. For practical application, it is highly attractive to incorporate TiO_2_ nanoparticles on flexible substrates. In the last years, for example, the incorporation of TiO_2_ nanoparticles on woven fabrics, such as cotton, silk, or wool, has been well investigated [8]. However, the anchoring of TiO_2_ photocatalyst on such flexible substrates is still challenging: the irregular shape of the fibers, very often, impedes the requirement for a sufficient interfacial adhesion of the photocatalyst to the fibers themselves, and therefore an adequate photocatalytic activity. Furthermore, many of the proposed fabrication process require post-deposition high-temperature treatments to promote crystallinity of TiO_2_, which are incompatible with the low chemical and thermal resistance of woven fabrics [9,10,11]. In this respect, the possibility to use also common paper as flexible substrate to anchor TiO_2_ nanoparticles has stimulated great attention from research and industrial communities, as paper has many advantages such as low cost, flexible, easy to handle, environmental friendly, and renewable [12]. Photocatalytic paper sheets indeed can find application for numerous purposes, i.e., medical and protective purposes, food packaging, filters, etc. The concomitant use of TiO_2_, in particular, may result in the fabrication of paper sheets with high efficient self-cleaning activity, having the capability to degrade adsorbed stains, bacteria, and volatile organic compounds (VOCS) transforming them into harmless CO_2_ and H_2_O molecules [13]. Therefore, a detailed study of the preparation process and application of the active coating is of significant importance in the manufacturing process of high quality and end products.

Furthermore, the successful application of TiO_2_-modified paper sheets can overcome the environmental drawbacks often associated with the recollecting and removal of free TiO_2_ nanoparticles suspended in treated water after the photocatalytic process. In this contest, several techniques such as sol–gel processing [14], liquid phase deposition [15], and chemical vapor deposition [16] can be employed to fabricate TiO_2_-based nanostructures. Among these preparation techniques, the relatively simple sol–gel method is very appealing in terms of cost effectiveness and better environmental impact. However, very often the sol–gel process requires high temperatures to produce highly crystalline TiO_2_, which generally assures better photocatalytic performance [17]. The formation of photocatalytic TiO_2_ nanoparticles at low temperature is highly desirable for any practical application involving the utilization of thermolabile materials, such as paper sheets. In addition, the destructive effect of strong acids frequently used in the sol–gel process to keep aqueous sols in the peptized state hinders such applications on thermolabile substrates. Daoud and his coworkers [7] reported on the fabrication of nanosized TiO_2_-modified paper by a hydrothermal process that involved relatively low temperature (97 °C). Recently, the blending of paper pulp with TiO_2_/sodium alginate nanocomposites was successfully applied to the fabrication of photocatalytic paper [18]. In this case, sodium alginate acted mainly as a template to control the nucleation and growth of TiO_2_ nanoparticles.

In this paper, we report the successful fabrication of photocatalytic paper obtained by introducing TiO_2_ hydrosol in the paper sheet production process. The TiO_2_ hydrosol was prepared at room temperature in aqueous medium using titanium butoxide as titanium source and the mild acetic acid as catalyst for the hydrolysis of the titanium precursor. An aging process was finally applied to obtain highly crystalline anatase TiO_2_ nanoparticles in the hydrosol. An efficient binding of the nanoparticles on the surface of cellulose fibers is an important factor that plays a key role for enhancing the photocatalytic properties of the modified paper sheets. Natural polysaccharides were extensively studied for this purpose. Sodium alginate is a kind of linear natural polysaccharide extracted from brown seaweed [19], and very recently it received considerable attention due to its good biodegradability, biocompatibility, and renewability. Considering its complete compatibility with the paper sheet-making process, and the presence of binding sites due to its carboxylate functional groups, sodium alginate was chosen for increasing the binding of the synthesized TiO_2_ nanoparticles over cellulose fibers. Note that the paper sheets fabricated in this work were obtained from the chemical treatment of agricultural Egyptian waste. Agricultural residues are used in many types of applications such as biomass energy, fertilizers, livestock feeding, and furniture. In Egypt, agriculture residues like rice straw, bagasse, and cotton stalks are produced in mass amounts [20] and they are currently used in the paper industry [21]. In this industry, paper with improved intrinsic properties obtained from agricultural waste is significantly very important in terms of control of secondary pollution and sustainability.

The physicochemical properties of the modified paper sheets with different TiO_2_ hydrosol loading content have been fully investigated using atomic force microscopy (AFM), X-ray diffraction (XRD), Fourier transmitted infrared (FTIR) and µ-Raman spectroscopy, and UV−Visible spectroscopy. As previously outlined, paper suffers some drawbacks in terms of low strength properties, and the effects of TiO_2_ loading content on the mechanical strength and barrier properties of TiO_2_-modified paper were investigated in detail. Finally, the photocatalytic activity of the modified paper sheets was verified by monitoring the photodegradation of methylene blue (MB) aqueous solution under UV light irradiation. The results showed that the proposed approach is undoubtedly a green and cost-effective process in terms of application, development, and preparation.

## 2. Materials and Methods

### 2.1. Materials

The bagasse fibers were kindly supplied from Quena Company of the pulp and paper industry (Cairo, Egypt). The chemical compositions of bagasse raw material were determined in accordance with Tappi standard methods, namely, lignin 20.40% (T-222 om-88), α-cellulose 41.50% (T 203 cm-99), hemicellulose content 27.20% (T-223 cm-84), and ash 1.80 % (T-211 om-02). All measurements were carried out in triplicate and the results are recorded as the mean value for each set of measurements. Titanium butoxide (≥ 99%) and glacial acetic acid were reagent grade (Alfa Aesar, Harverhill, MA, USA). Sodium alginate (*M*v = 1.2 × 105, *μ* = 280 mPa.s) was acquired from the Shanghai Chemical Reagent Co. (Shanghai, China). Deionized water was used throughout the study. All reactants were used as received. 

### 2.2. Preparation of Nanocrystalline TiO_2_ Hydrosol by Aging at Room Temperature

Ti(OBut)_4_ and glacial acetic acid were carefully mixed in a volume ratio of 1:2, respectively, under magnetic stirring at room temperature. After 10 min of vigorous stirring, the acidic dispersion containing titanium was added dropwise to 100 mL of deionized water. The mixture was stirred at room temperature for 24 h, and then the aqueous white dispersion was stored in dark without stirring at room temperature and atmospheric pressure. After five days, the white suspension turned into a yellowish translucent colloidal TiO_2_ hydrosol. The final pH was 2.25 ± 0.05. TiO_2_ powders were precipitated from the hydrosol by shifting the pH from acidic to neutral through the addition of a 10%w/w sodium carbonate solution. The precipitate TiO_2_ nanoparticles were collected by centrifugation at 6000 rpm (ThermoScientific IEC CL31R Multispeed centrifuge, Waltham, MA USA), washed three times with deionized water, and finally oven dried at 45 °C overnight.

### 2.3. Preparation of Photocatalytic Paper Sheets

#### 2.3.1. Paper Sheet Preparation

Bagasse raw material was treated chemically to remove lignin and extract cellulose fibers: bagasse raw materials were exposed to soda kraft pulping process using 80 g/L total active alkali (NaOH + Na_2_S) and 12% sulfides (Na_2_S/NaOH + Na_2_S) at a 1:3.5 liquor ratio for 120 min at 160–170 °C. The pulp was isolated from black liquor by several washing. Three stages of bleaching process were carried out: (1) chlorine dioxide (D0-Stage) by adding 10% ClO_2_ based on dry oven pulp with 10% H_2_SO_4_ for adjusting pH at 60–65 °C for 1 h, (2) oxygen stage (EO-Stage) by adding 10% (H_2_O_2_ + O_2_) in presence of 10% H_2_SO_4_ at 60–65 °C for 1.5 h, and (3) chlorine dioxide (D1-Stage) 5% ClO_2_ based on dry oven pulp with 10% H_2_SO_4_ at 60–65 °C for 4 h. The bleached kraft bagasse pulp was washed several times and subjected to refinery disks to 40-45° SR. 

Paper handsheets of a basis weight of 80 g/m^2^ were prepared according to the S.C.A standard, using the model S.C.A sheet former (AB Worentzen and Wettre). The weight of oven dry pulp used for every sheet was approximately 1.8 g. The pulp suspension was then diluted by 5–7 L of water. After a stirring phase (air injection avoids aeration), the suspension was filtrated on a wire. In the machine, a sheet of 165 mm diameter and 214 cm^2^ surface area was obtained. The wet sheet was pressed for 4 min using a hydraulic press, and then it was collected on blotting paper, and dried using a rotating cylinder or drum at 105 °C for 2 h. 

#### 2.3.2. Fabrication of TiO_2_ Hydrosol/Sodium Alginate Hybrid Nanocomposite on Paper Sheets

Sodium alginate solution was prepared by dissolving the sodium alginate (2% w/v) in distilled water while stirring for 2 h. Then, after complete dissolution, different concentrations of TiO_2_ nanoparticles (0.125, 0.25, 0.50, and 1%w/v) were added and stirred at the constant stirring condition of 3000 rpm for 1 h. The solution was further sonicated for 30 min. The prepared sheets were coated with different concentrations of TiO_2_/sodium alginate nanocomposite solution with thickness 120 µm in an applicator, and then dried on air at room temperature. All paper samples were conditioned before testing at temperature of 23 ± 1 °C and 50 ± 2% relative humidity for 24 h according to the standard ISO 187-1977.

### 2.4. Characterization of TiO_2_ Hydrosol and TiO_2_-Modified Paper Sheets

X-ray diffraction (XRD) patterns were recorded on a Siemen D5000 X-ray diffractometer, using Cu kα (λ = 1.5406Ǻ) radiation and operating at 40 kV and 30 nA. 

UV−Vis diffuse reflectance spectra were recorded by a Jasco double-beam V660 UV−Vis spectrophotometer (Tokyo, Japan) in reflectance mode, equipped with a 60mm integrating sphere and BaSO_4_ as standard diffuse reflectance material.

Transmission Electron Microscope (TEM) for TiO_2_ hydrosol was carried out using a high-resolution JEOL- JEM 2100 (Tokyo, Japan).

Environmental scanning electron microscopy (ESEM) and Energy-dispersive X-ray spectroscopy (EDAX) investigations were carried out using a Quanta FEG-250 microscope (Waltham, MA, USA) at a voltage of 20 kV. 

Atomic force microscopy (AFM) was carried out using a Dimension 3100 atomic force microscope equipped with a NanoScope IIIa controller (Veeco, Santa Barbara, CA, USA) operating in tapping mode. Silicon nitride (TESP) probes with a resonant frequency of ~300 kHz and a nominal spring constant of 20/80 Nm were employed (Veeco, Santa Barbara, CA, USA). A scan rate of 0.3–1 Hz was employed at a resolution of 512 pixels/line. 

FTIR measurements were carried out by an Alpha FT-IR spectrometer (Brucker Optics, Ettingen, Germany) equipped with exchangeable sampling modules. The spectra were collected as the average of at least 200 scans at a resolution of 4 cm^−1^ in the frequency range 4000–500 cm^−1^. An external reflection module was employed for the spectroscopic investigation of the samples analysing a few spots (~3 nm diameter) for the sake of comparison, controlling, and monitoring the sampling areas with an integrated video camera.

Raman spectra were collected with a µ-Raman spectrometer (Renishaw RM 2000, Gloucestershire, UK) operating in the backscattering configuration, and equipped with a Peltier cooled charge-coupled device (CCD) camera in conjunction with a Leica optical microscope with a 50× objective. For data analysis, Stokes lines were considered. Incident light was focused on each sample through the optics. Measurements were performed using the 785 nm emission line of a laser diode.

### 2.5. Evaluation of Photocatalytic Activity of TiO_2_ Hydrosol and TiO_2_-Modified Paper Sheets 

The photocatalytic activities of TiO_2_ nanoparticles were estimated by the degradation of methylene blue (MB) in aqueous solution at room temperature using the method described in our previous paper [22]. Briefly, 50mg of the powder photocatalysts were ultrasonically dispersed in 100 mL of 10^−5^M MB aqueous solution. Before irradiation, the sample was magnetically stirred and kept in the dark for 60 min to ensure the adsorption–desorption equilibrium of MB on the surface of the photocatalyst. The suspension was then exposed to either a 39W UV lamp with two emission wavelengths (254 and 365 nm). The change in dye concentration was evaluated by measuring the absorbance maximum of MB at 665 nm. Therefore, the degradation efficiency was calculated using the formula (C_0_−C_t_/C_0_) × 100, where C_0_ is the initial concentration of the original MB aqueous solution, and C is the concentration of MB solution after every given time intervals.

TiO_2_-modified paper sheets were cut in small pieces (3 × 3 cm^2^): each piece was immersed in 50 mL of 10^−5^M MB aqueous solution and maintained in the dark for 120 min. The stained TiO_2_-modified paper sheets were dried in dark, and then they were exposed for a given period of time to a 39 W UV lamp at excitation wavelength of 365 nm. The residual MB on TiO_2_-modified paper sheets was assessed by UV–Vis measurements, fixing the samples on the hole of the integrating sphere and recording the absorbance variation of MB on the paper surface at certain time intervals 

### 2.6. Mechanical and Barrier Properties for Photocatalytic Paper Sheets 

Strength properties (tensile, burst, and tear indices) were determined according to Tappi standard methods. Average and standard deviation for all measurements was calculated based on 5 replicates for each sample. Tensile strength testing was measured according to TAPPI (T494-06) standard method using a universal testing machine (LR10K; Lloyd Instruments, Fareham, UK) with a 100-N load cell at a constant crosshead speed of 2.5 cm/min. Strips of 10 cm long and 15 mm wide were used in tensile strength test and the span was set at 10 cm. Tear resistance was carried out with an Elmendorf-type tear tester (Thiwing-Albert Instrument Co, Philadelphia, PA, USA) using TAPPI (T414-04) standard method. Burst strength was carried out according to TAPPI Standard test method (T403 om-10) using Mullen Testers (Chicopee, MA, USA). 

Air permeability of the paper sheets was measured according to ISO 5636, using a Bendtsen smoothness and porosity tester made in Denmark (Andersson and Sorensen, Copenhagen). The air permeability was determined as the rate of air flow between the paper surface and two concentric, annular metal rings, which were applied to the paper samples of area 2 cm^2^, under ambient air conditions and standard pressure, while applying a vacuum of 2.5 kPa. The time of measurement was 5 min for each sample, and the average air permeability was calculated from at least five measurements. Air permeability is partially dependent on the uniformity and porosity of the coating layer, and it is a physical parameter that determines the resistance of the paper to air flow.

Water vapor permeability (WVP) was carried out according to the standard ASTM E96/E96M-10. Coated paper sheets were conditioned at 25 °C and 50% RH for 24 h, and then they were used to hermetically cover aluminum cups containing 5g of anhydrous calcium chloride. The coated side was that facing the humidified side; the WVP was calculated using

WVP = g x / t A ΔP

where (g/t) = the slope of the plot between weight gain(g) and time (t), x = the average thickness of the papers, A = the permeation area, and ΔP = the partial water vapor pressure difference between the ambiance in the cup (0%RH) and the saturated sodium chloride solution (75%RH ≈ 2385 Pa). Three-fold determinations were performed for all samples, and average WVP was measured. Standard deviations (SD) were calculated for all measurements.

## 3. Results and Discussion

### 3.1. Characterization of TiO_2_ Nanoparticles

**Figure 1:**
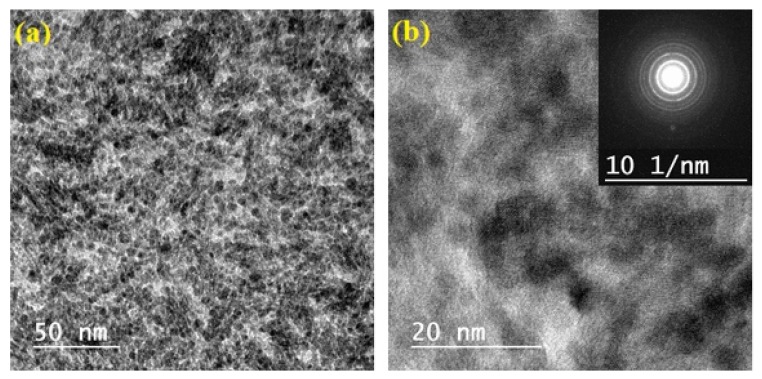
(**a**) TEM and (**b**) HRTEM images of TiO_2_ nanoparticles; Inset: selected area electron diffraction (SAED) images of TiO_2_ nanoparticles.

Transmission electron microscopy (TEM) images of TiO_2_ hydrosol are shown in Figure 1. TEM investigations pointed to the formation of quasi-spherical shape TiO_2_ nanoparticles with slight agglomerations, and an average size of about 3.8 nm. These results were comparable with those obtained using low-temperature sol–gel methods [23,24]. The image with higher magnification is also shown in Figure 1b: lattice fringes indicated that the particles are nanocrystalline with anatase phase, which is also confirmed by X-ray diffraction. The selected area electron diffraction (SAED) confirmed that TiO_2_ nanoparticles are in the anatase phase with good crystallinity (inset Figure 1b): the rings can be clearly indexed to diffraction from the (101), (004), (200), (105), (211), and (204) planes of TiO_2_ anatase.

**Figure 2:**
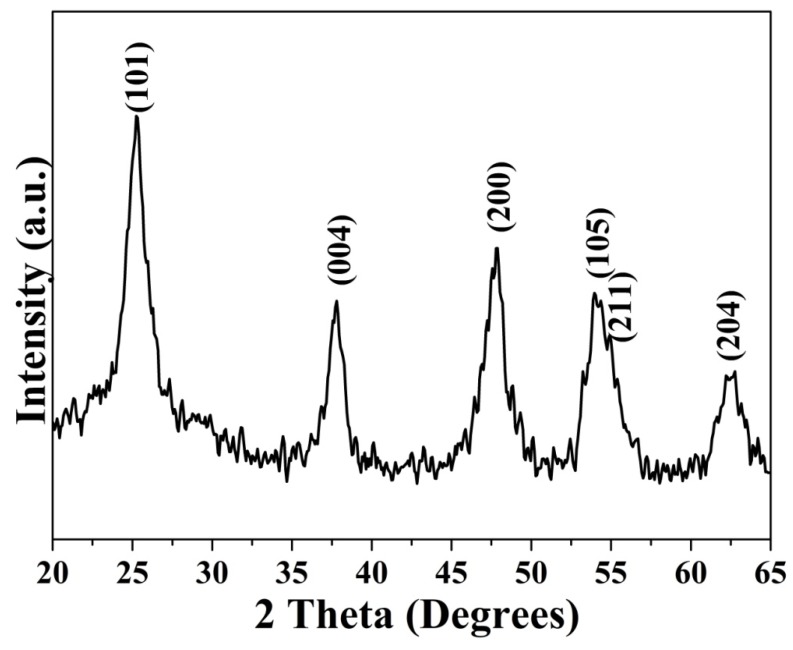
XRD pattern of TiO_2_ nanoparticles.

The structure of TiO_2_ nanoparticles and their crystallinity were further confirmed by X−ray diffraction (XRD) (Figure 2). All the diffraction peaks in the XRD spectrum were related to the characteristic peaks of TiO_2_ in the anatase phase (JCPDS file 73-1764). The diffraction peaks indeed appeared at 2θ = 25.23°, 37.71°, 47.72°, 54.16°, 55.32°, and 62.54°, and they were well indexed to the corresponding tetragonal crystal planes (101), (004), (200), (105), (211), and (204), respectively. The XRD peak broadening was used to evaluate the size (D_hkl_) of the crystal domains, and it was calculated from the widths at half maximum height (B) using the Debye−Scherrer equation D_hkl_ = $\frac{K\lambda }{B\cos\theta }$, where λ is the X-ray wavelength of the incident beam (1.5406Å), θ is the Bragg angle, and K is a constant, approximately equal to 0.9, related to the domain shape. The average crystallite size of the synthesized TiO_2_ nanoparticles was calculated as ~7.09 nm (Table 1).

**Figure 3:**
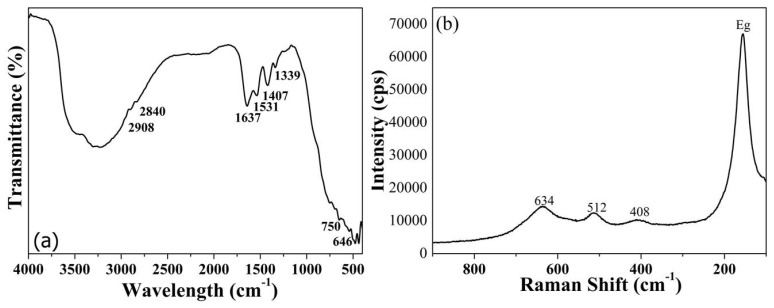
(**a**) FTIR and (**b**) Raman spectra of TiO_2_ nanoparticles.

The chemical structure of TiO_2_ nanoparticles was investigated by FTIR spectroscopy to obtain information on the presence of certain functional groups on their surfaces. The FTIR spectrum showed the peaks corresponding to TiO_2_ (Figure 3a): the broad absorption in the range 800 to 400 cm^−1^ derived from the contribution of the anatase TiO_2_, and the peaks between 750 and 646 cm^−1^ were assigned to the Ti–O–Ti stretching vibrations [25]. The band at 1408 cm^−1^was assigned to TiO_2_ lattice vibrations [26]. The strong and broad absorption at 3000–3600 cm^−1^ and the peak at 1637 cm^−1^ are related to the O−H stretching mode and O–H bending vibrations, respectively, of hydroxyl groups, deriving from the presence of adsorbed water molecules on the surface of TiO_2_ nanoparticles [27]. The FTIR spectrum showed also the absorption bands related to the vibrational modes of organic residual species, such as carboxylate and alkane groups. The two weak absorptions at 2908 and 2840 cm^−1^ are characteristic of stretching vibrations of methyl groups. The peak at 1531 and 1339 cm^−1^ could be attributed to carboxyl (C=O) and methylene groups. In this case, the alkane and carboxylic groups could arise from titanium butoxide and acetic acid used as precursors during the synthesis.

In Figure 3b, the Raman spectrum of synthesized TiO_2_ nanoparticle is reported. According to factor group analysis, anatase has six Raman active modes (A_1g_ + 2B_1g_ + 3E_g_). The Raman spectrum of an anatase single crystal was first investigated by Ohsaka, who concluded that the six allowed modes appear at 144 cm^−1^ (E_g_), 197 cm^−1^ (E_g_), 399 cm^−1^ (B_1g_), 513 cm^−1^ (A_1g_), 519 cm^−1^ (B_1g_), and 639 cm^−1^ (E_g_) [28]. The synthesized TiO_2_ nanoparticles showed the characteristic Raman active modes of the anatase phase; however, a significant shift of the E_g_ peak (155 cm^−1^) was observed. The observed Raman shift is indicative of the presence of TiO_2_particles with size in the nm range. Choi et al. [29] reported that when the particle size decreases to the nanometer scale, a volume contraction occurs within the nanoparticles, which leads to an increment in the force constants: as a consequence, the observed Raman bands shift towards higher wavenumbers. 

**Figure 4:**
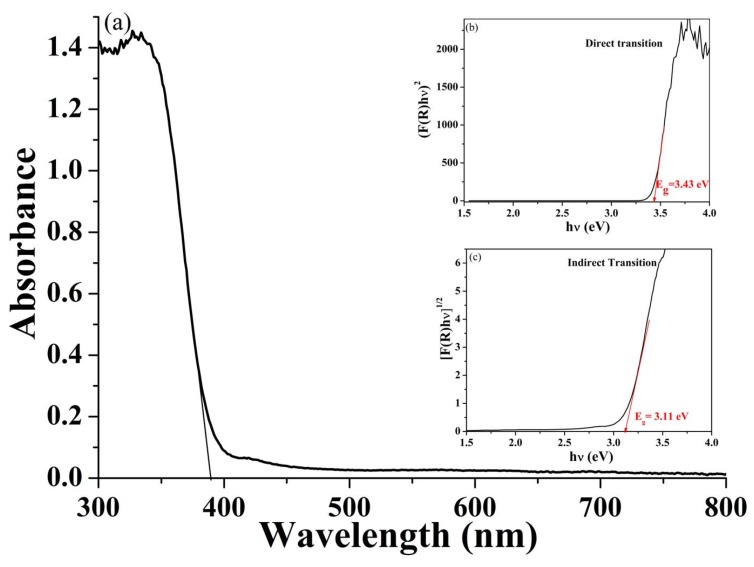
(**a**) UV−Vis diffuse reflectance spectrum of TiO_2_ nanoparticles; (**b**) (F(R)hν)^2^ vs. energy of the adsorbed light plot considering TiO_2_ as a direct semiconductor; and (**c**) (F(R)hν)^1/2^ vs. energy of the adsorbed light plot considering TiO_2_ as an indirect semiconductor.

The optical band gap of TiO_2_ nanoparticles was obtained by diffuse reflectance spectroscopy in the UV−Vis range (Figure 4a). As expected, TiO_2_ nanoparticles exhibited a strong light absorption below 400 nm with a maximum at around 326 nm, attributed to a band-to-band TiO_2_ transition [30]. The optical band gap energy (E_g_) was determined using the Tauc equation [(αhν)^n^ = B(hν-Eg)], where α is the optical absorption coefficient, hν is the incident photon energy, E_g_ is the band gap energy, B is a constant, and n is a number that depends on the nature of the transition being n=2 for allowed direct transitions and n = 1/2 for indirect ones [31]. As the E_g_ of a semiconductor is usually determined from diffuse reflectance spectra, the corresponding reflectance spectra were transformed to absorption ones by applying the Kubelka–Munk function and obtaining the following equation; (F(R_∞_)hν)^n^ = B(hν − E_g_), where R_∞_ = R_sample_/R_standard_ is the reflectance of an infinitive thick specimen [32]. Bulk TiO_2_ is an indirect semiconductor; however, in “nanoparticle systems”, the lattice periodicity could be lost over the length scale of the nanoparticle size, so direct transitions could in principle take place [22]. To establish the nature of the band-to-band transition in TiO_2_ nanoparticles, the absorption data were fitted to a Tauc plot considering both direct and indirect bandgap transition. The corresponding spectra showed the steep and linear increase of light absorption upon increasing energy characteristic of semiconductor materials [33,34]. Considering TiO_2_ as an indirect semiconductor, a band gap energy value of allowed indirect transition of ~3.11 eV was obtained (Figure 4b), which was less than that reported for the bulk [35]. When TiO_2_ was considered as a direct semiconductor, a sensibly higher E_g_ was obtained (E_g_ = 3.43 eV). This value seemed to be more realistic, as already reported in the literature for anatase nanoparticles [30,36]. The observed discrepancy could be explained by considering that the band gap of semiconductors was found to be grain size-dependent [37]: it is well accepted that E_g_ generally increases as the grain sizes decrease and the absorption edge was shifted to a higher energy (blue shift). This consideration together with our experimental results suggests that in the case of synthesized TiO_2_ nanoparticles, direct transition is more favorable compare to indirect one. The occurrence of direct bandgap transitions is generally considered positive because they imply very strong electronic absorption as well as emission, and therefore better photocatalytic performances can be reasonably expected [22,30].

### 3.2. Photocatalytic Activity of TiO_2_ Nanoparticles

**Figure 5:**
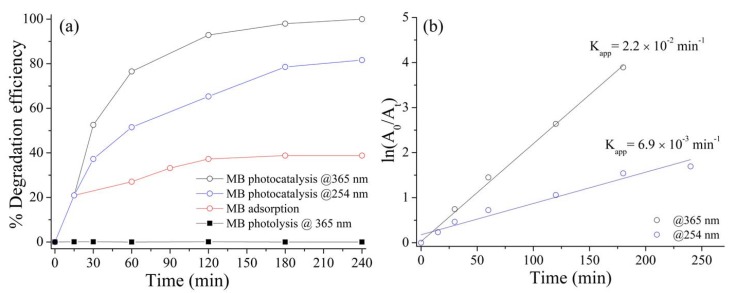
(**a**) Time evolution of photocatalytic decomposition efficiency of methylene blue under different UV wavelength irradiation; (**b**) kinetics of photocatalytic decomposition efficiency of methylene blue under different UV wavelength irradiation.

We tested the activity of synthesized TiO_2_ hydrosol for methylene blue (MB) dye, because, generally, colored dyes are very common pollutants in industrial wastewater and they are difficult to remove by biological treatment methods [38]. In this study, the photocatalytic activity of synthesized TiO_2_ nanoparticles was obtained from the % of degradation rate versus time, as well as the kinetic data using MB as representative pollutant model. For the photocatalysis experiments, 50 mg of TiO_2_ NPs was suspended in a proper volume of MB solution 10^−5^ M, and the progress of the photocatalytic reaction was monitored by the gradual decrease of MB absorbance at 665 nm with time. All the photocatalytic experiments were carried out using an UV lamp having two emission wavelengths (254 and 365 nm), as the optical characterization did not revealed any significant absorption in the visible range (Figure 5a). The corresponding plot of absorbance variation versus time (Figure 5b) showed a profile of exponential nature, thus indicating that the photocatalytic reaction of MB obeyed to the pseudo-first-order kinetic: the apparent pseudo-first-order rate constant, k, was calculated from the slope of the straight line plot ln(A_0_/At) vs time.

In terms of photocatalytic efficiency, degradation under 365 nm UV light showed the highest activity, being almost complete after 240 min of irradiation, whereas under 254 nm UV light, the degradation efficiency reached the 81% after 240 min. A similar trend was confirmed for the pseudo-first-order rate constants, k: under 365 nm UV light irradiation, the rate constant k is approximately one order of magnitude greater than the value found under 254 nm UV irradiation. Nevertheless, under dark a slight decrease in the absorbance of MB was observed, meaning the absorption of the dye on the surface of the TiO_2_ nanoparticles dispersed in solution. However, the absorption reached a constant value after about 120 min with a degradation efficiency significantly lower than those obtained under UV light, thus confirming that light is an essential requirement for the photodegradation of MB. Moreover, photodecomposition of MB under the irradiation of two different UV wavelengths in the absence of catalyst was negligible.

### 3.3. Structural and Morphological Characterization of TiO_2_−Modified Paper

Four kinds of hybrid paper having different content of TiO_2_ hydrosol (1.0, 0.5, 0.25, and 0.125%) were prepared. All the prepared TiO_2_ modified paper sheets were white and bright. 

**Figure 6:**
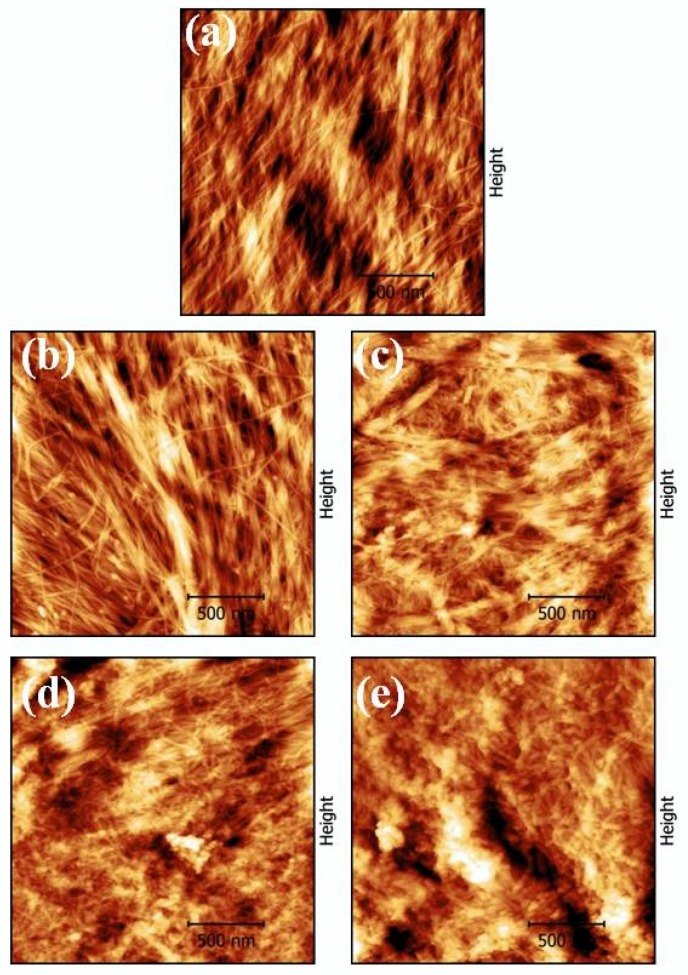
Atomic force microscopy (AFM) topography images of (**a**) blank paper sheet and (**b**–**e**) paper sheets modified with 0.125, 0.250, 0.500, and 1.0 % of TiO_2_, respectively.

The influence of surface modification on the topography and roughness of the paper sheets was investigated by AFM microscopy (Figure 6). AFM analysis was first performed on blank paper and then compared to the modified samples with different amounts of TiO_2_ loadings (0.125–1.0%). As shown in Figure 6, the AFM investigation pointed to a very similar microstructure between the different papers with the cellulose fiber being interconnected and forming a porous structure. However, AFM analysis revealed that the morphology of the cellulose fibers was changing depending on the percentage of TiO_2_ loading. Indeed, the morphological features of blank paper (Figure 6a) showed that it was characterized by smooth and regularly ordered fibers with diameter in the range of 10 to 20 nm and a surface root mean square (RMS) of ~5.49 nm. After modification with TiO_2_ hydrosol, spherical particles with size of ~20–35 nm appeared on the surface of cellulose fibers. The density of the spherical particles increased with the increase in TiO_2_ loading, resulting also in the increment of the surface roughness of modified paper sheets. In particular, the surface RMS values were approximately 5.27, 5.73, 7.30, and 13.00 nm for 0.125, 0.25, 0.5, and 1.0% TiO_2_-modified papers, respectively. In the case of 0.5 and 1.0% TiO_2_-modified paper sheets, the AFM analysis revealed that TiO_2_ coating was uniformly deposited on the surface of the paper, with some areas showing TiO_2_ aggregation (Figure 6d,e). As suggested by Jaksik et al. [39], however, these clusters of TiO_2_ could be effective in increasing the available surface area on the surface of the fiber, thus contributing to increase the final photocatalytic activity.

**Figure 7:**
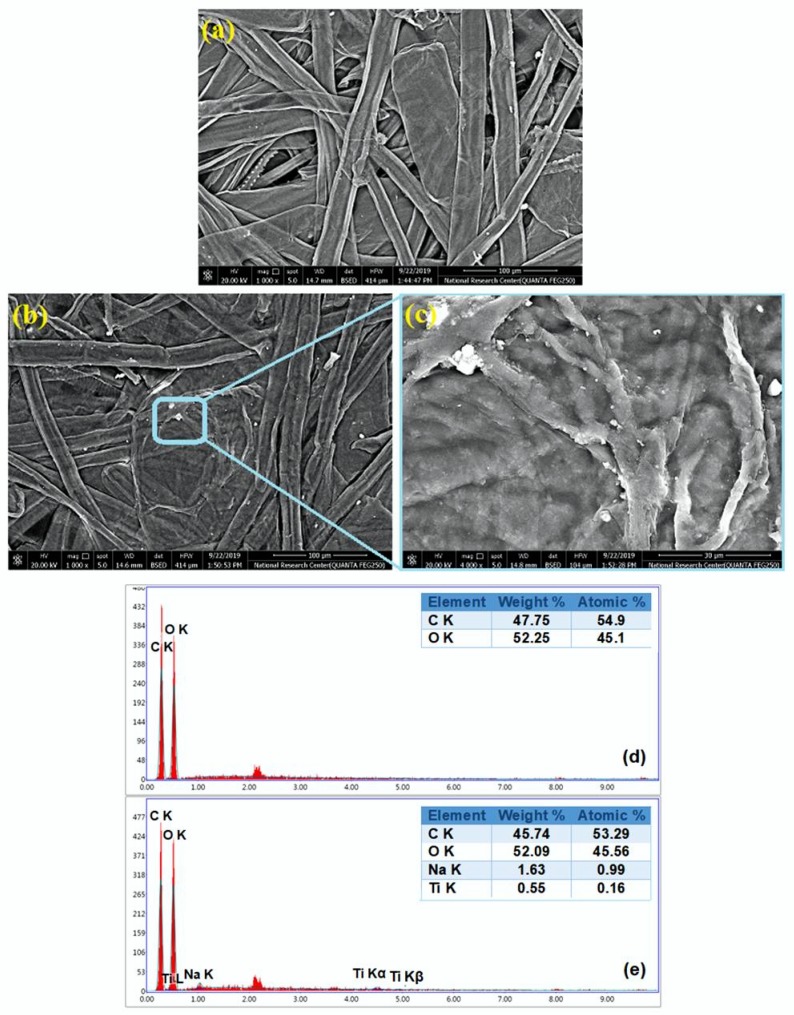
SEM images of (**a**) blank paper sheet, and (**b**,**c**) 1.0% TiO_2_-modified paper sheet, Energy-dispersive X-ray spectroscopy (EDAX) analysis of (**d**) blank paper sheet, and (**e**) 1.0% TiO_2_-modified paper sheet

The morphological properties of the paper coated by TiO_2_ hydrosol were further examined by SEM (Figure 7). In case of blank paper, the cellulosic matrix appeared open, porous, and randomly dispersed (Figure 7a), whereas in the 1.0% TiO_2_-modified paper sheet, the porous structure was lost and the cellulosic fibers were uniformly coated and cross-linked to each other (Figure 7b). SEM investigation confirmed the AFM results: TiO_2_ nanoparticles are dispersed on the surface of cellulosic matrix, although some aggregates occurred (evidenced by red arrows in Figure 7c), probably due to some retention effects of sodium-alginate on TiO_2_ nanoparticles [40]. As expected, the EDAX analysis revealed that in the case of blank paper the most intense signals are due to C and O: the main constituents of the paper sheets (Figure 7d). In the 1.0% TiO_2_-modified paper sheet, the presence of Na peak is due to sodium alginate, whereas the existence of TiO_2_ nanoparticles is confirmed by the appearance of Ti peaks (Figure 7e).

**Figure 8:**
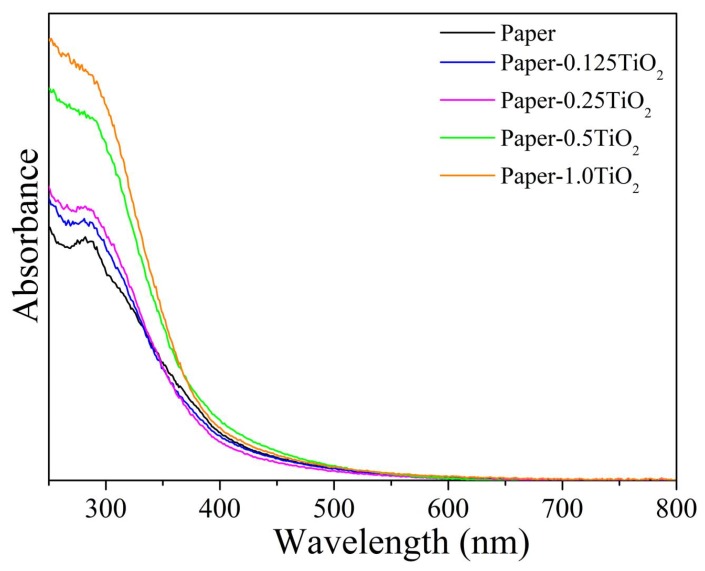
UV–Vis diffuse reflectance spectra of TiO_2_-modified paper sheets with different loading of TiO_2_ hydrosol.

The absorption spectra of blank and modified paper with different amount of TiO_2_ are shown in Figure 8. As expected, all the TiO_2_-modified paper sheets showed higher absorption ability in the UV range respect to unmodified paper, and the absorbance in the UV region increased upon increasing the amount of TiO_2_ loading in the paper sheets. Consequently, the incorporation of relative higher TiO_2_ loading (0.5% and 1%) had the effect to absorb higher fraction of photons in the UV region, and the resulting modified paper sheets were supposed to show good UV blocking property [41].

**Figure 9:**
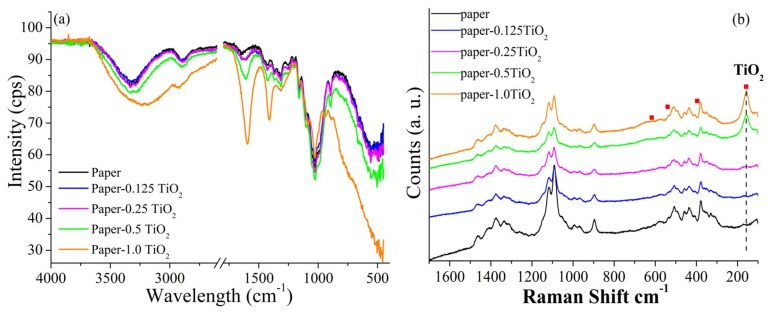
(**a**) FTIR spectra and (**b**) Raman spectra of TiO_2_-modified paper sheets with different loading of TiO_2_ hydrosol.

The FTIR spectra of paper and TiO_2_ modified paper are reported in Figure 9a. The FTIR spectrum of the unmodified paper showed the characteristic absorption peaks of cellulose. In particular, it was characterized by an intense absorption band in the 3600 to 3100 cm^−1^ range, associated with the stretching of O−H bond of the hydroxyl groups of cellulose. A peak at 1645 cm^−1^ was also indicative of the presence of interstitial or adsorbed water in the cellulose structure. The absorption in the 3000 to 2800 cm^−1^ range was due to the C−H stretching vibration, whereas that in the 1450 to 1350 cm^−1^ range was due the asymmetric and symmetrical deformation of CH_2_ and C−H groups. Finally, the complex and intense absorption in 1300–900 cm^−1^ range represented the fingerprint of cellulose and it was mainly associated with stretching mode of C–O–C (1060 cm^−1^) and C−O (1028 cm^−1^) bonds in the cellulose framework. The position of these bands was affected by inter- and intramolecular hydrogen bonds, and therefore it was strictly related with changes in the chemical surface groups [42]. When TiO_2_ was introduced into the paper sheet a general change of O−H absorption peaks in the corresponding FTIR was observed. In particular, as compared to the unmodified paper sheet, both the O−H stretching and O–H bending absorption peaks shifted towards lower wavelengths and increased significantly their intensity. This result could be indicative of a strong interaction occurred at the interface between the –OH groups of cellulose and Ti-O bonds in TiO_2_ [43]. Upon increasing the TiO_2_ loading, the intensity of the peaks at approximately 1300–900 cm^−1^ for TiO_2_-modified paper sheets, due to C–OH stretching (1060 cm^−1^) and C–O–C bending vibrations (1163 cm^−1^), gradually decreased in comparison to the peaks in unmodified paper sheet because of the presence of TiO_2_ nanoparticles on the surface of the cellulose fibers [28,44]. A similar phenomenon was observed for the C–H stretching vibration (2910 cm^−1^). Due to presence of TiO_2_ nanoparticles, the C–H in-plane stretching was blocked as result of steric hindrance. As reported by Mohamed et al. [45], the strong interaction between the cellulose and TiO_2_ nanoparticles can be indicative of a better durability of nanoparticles within the paper sheets and hence it is expected a low detachment tendency of nanoparticles within the cellulosic matrix.

In Figure 9b, the Raman spectra of the blank paper and TiO_2_-modified paper sheets are reported: in all the spectra, the characteristic fingerprint region of cellulose (250–1500 cm^−1^) was clearly observed. This region was characterized by the contributions of CH and CH_2_ bending, CH wagging, OH rocking and bending, and CO and COC stretching [46]. The spectra of TiO_2_-modified paper furthermore confirmed the incorporation of the TiO_2_ nanoparticles on the paper sheets: in particular, the E_g_ peak at 157 cm^-1^ was particularly evident, and its intensity increased as the loading of TiO_2_ nanoparticles in the paper increased. Furthermore, in analogy to what was observed for TiO_2_ hydrosol (Figure 3b), the E_g_ Raman peak shifted towards higher values.

### 3.4. Strength and Barrier Properties of Modified Paper Sheets

**Figure 10:**
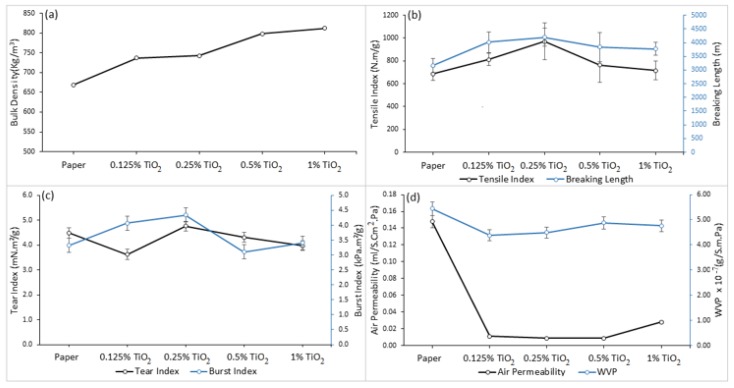
(**a**) Bulk density, (**b**) tensile and breaking length, (**c**) tear and burst index, and (**d**) air and water vapor permeability for paper sheets modified by TiO_2_ nanoparticles.

Paper bulk density elucidates the relation between the thickness of paper and its grammage. Paper sheets with high bulk densities are in general thick, airy, light, and opaque. As expected, the bulk density of the modified paper sheets increased linearly upon increasing the amount of TiO_2_ loading (Figure 10a).

Our investigation indicated that in general TiO_2_-modified paper sheets showed better mechanical performance respect to unmodified paper. In the case of the tensile index, which is a measure of inherent strength of paper, it was found that the addition of TiO_2_ nanoparticles led to an increase in the measured tensile index, which reached its maximum (971.37 N m/g) for the 0.25%TiO_2_-modified paper sheet (Figure 10b). In analogy, the presence of TiO_2_ nanoparticles was responsible for the observed increment in the measured breaking length. Also, modification of paper fibers with TiO_2_ increased the burst index of modified paper considerably (Figure 10c). The reason for the favorable mechanical performance of the TiO_2_-modified paper sheets is probably due to the abundant hydroxyl groups on the surface of TiO_2_ that can form hydrogen bonds with the hydroxyl groups in both sodium alginate and cellulose, thus improving the adhesion between cellulosic fibers and therefore the fiber–fiber bonding. However, the effect of fiber bonding strength on the tear index is complicated (Figure 10c). In the case of a network with strong fiber bonding, the tear index was decreased by increasing the fiber bonding, whereas in the case of a network with weak fiber bonding, the tear index was in direct relationship with the fiber bonding. This behavior may be related to the particle number and particle size of nanocomposite [47].

Air and water vapor permeability of the modified paper sheets were measured as well and compared with those of unmodified paper. Figure 10d indicates that the air permeability decreased drastically when the TiO_2_ hydrosol was added onto paper sheet. The observed decrease could be explained by the fact that TiO_2_ nanoparticles caused the fiber matrix to become less porous to some extent: as observed from SEM characterization, the blending of TiO_2_ nanoparticles with sodium alginate is responsible for the formation of an homogeneous network on the paper sheet that strongly reduces the voids in the paper matrix, producing a strong and smoother surface, and improving the air resistance. The water vapor permeability (WVP) of modified paper sheets, which is the volume of water vapor passing through a paper matrix per unit area and time under definite conditions, was measured. From Figure 10d, the treatment of paper sheets with TiO_2_ hydrosol caused a decrease in the water vapor permeability compared with the control sample. This may be attributed to a less hydrophilic character of the modified paper sheets that caused less water penetration through the paper network.

TiO_2_ nanoparticles can be considered effective in enhancing the mechanical properties of the modified paper sheets, as the resistance and permeability of the modified paper are strongly dependent on the amount of TiO_2_ loading.

### 3.5. Photocatalityc Properties of TiO_2_-Modified Paper

**Figure 11:**
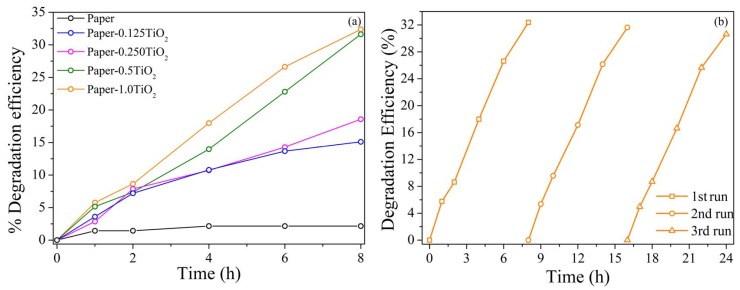
(**a**) Time evolution of modified paper with different loading of TiO_2_ hydrosol on photocatalytic decomposition efficiency of methylene blue under 365 nm UV light irradiation. (**b**) Time evolution of a 1.0% TiO_2_-modified paper sheet on the photocatalytic decomposition efficiency of methylene blue under 365 nm UV light irradiation after three consecutive cycles.

Based on the experimental results reported in the previous section, the photocatalytic activity of TiO_2_-modified paper sheets was evaluated by monitoring the degradation oxidation of MB under 365 nm UV light irradiation. The photocatalytic activity of an unmodified paper sheet was also evaluated as reference. As depicted in Figure 11a, the unmodified paper sheet showed negligible photocatalytic activity, confirming that the photocatalytic degradation of MB dye did not occur without the incorporation of the photocatalyst in the paper sheet. Therefore, the direct photolysis of MB was negligible in this study. Moreover, the degradation efficiency was increased by adding a greater percentage of photocatalyst to the paper sheets: the best results were therefore obtained in the case of the higher TiO_2_ loadings (1.0 and 0.5%) with a degradation efficiency of 32.3% and 31.6% respectively, after 8 hours of UV light irradiation. The lower efficiency on the opposite was observed for the paper loaded with only 0.125% of TiO_2_. In analogy, Rehim et al. [18] reported that the rate of the photocatalytic reaction increased upon increasing the concentration of TiO_2_/sodium alginate nanocomposite to the paper pulp, reaching the better performance (42% of COD removal) for the sample loaded with 20 wt.% nanocomposite. When the commercial TiO_2_ particles loaded on carbon fibers were used in the preparation of photocatalytic paper, a methyl orange photodegradation efficiency of ~80% was obtained after 7h of UV irradiation, which is higher than the photocatalytic activity showed by TiO_2_ particles directly loaded on cellulose fibers [48]. The better performances in this case were attributed to carbon fibers whose presence enhanced charge separation hindering the electron–hole pair’s recombination. However, note that the efficiency of the photocatalytic process is sensibly reduced when TiO_2_ is immobilized on paper sheets compared with the results obtained for TiO_2_ suspended in aqueous solution (Table 2). It was largely accepted that the immobilization of a photocatalyst on solid support often implies a decrease in the efficiency of the photocatalytic process because of a loss of the available catalytically active surface, a more difficult exchange with solution, and/or introduction of ionic species [49,50]. Rachel et al. [51], for example, compared the photocatalytic efficiency of TiO_2_ immobilized on various supports with the photocatalytic efficiency of TiO_2_ Degussa P25 in suspension, for the degradation of 3-nitrobenzenesulfonic acid (3-NBSA) and 4-nitrotoluenesulfonic acid (4-NTSA). They found that in all cases the photocatalytic activity of the immobilized TiO_2_ was significantly reduced. In their study, they concluded that the loss of efficiency of the immobilized TiO_2_ could not be attributed solely to the reduction of the active surface, but also to the presence of ionic species that favored the charge recombination processes. Despite the loss of efficiency, however, the immobilization of TiO_2_ on solid support such as paper sheets is still attractive because it offers a lot of advantages in terms of long-term and environmental safe stability (avoiding leaching of TiO_2_ nanoparticles in solution, for example) [52]. From an economically point of view, it is also attractive because it allows the simple recovery and reuse, eliminating the need of a postprocess filtration step to remove the photocatalyst nanoparticles from the treated water [53].

The stability of the modified paper sheets have been evaluated by carrying out three consecutive runs of photocatalytic degradation of MB for the sample loaded with the 1.0% of TiO_2_ hydrosol. After each cycle, the modified paper was washed very quickly with distilled water, blow dried, and re-immersed in a fresh dye solution. The experimental results are reported in Figure 11b. The 1.0% TiO_2_-modified paper showed moderately stable photocatalytic activity after three consecutive photocatalytic experiments. Moreover, the results revealed that the modified paper still possessed significant photocatalytic activity after three consecutive cycles of UV illumination, as the degradation efficiency of the modified paper decreased slightly from 32 to 27%. One possible reason for the observed decrement is found in the negative effects of dye accumulation on the pores of paper, which could be responsible for the coverage of catalyst surface: the intensity of light reaching the surface of catalyst nanoparticles was reduced, thus lowering the number of the photogenerated hole−electron pairs [54]. On the other hand, the degradation of paper after its continuous immersion in water could not be ruled out, which could cause the loss of some TiO_2_ nanoparticles, and therefore a slight decrease in the photocatalytic efficiency [40,48].

## 4. Conclusions

Photocatalytic paper sheets with improved photocatalytic activity were fabricated by adding different amounts of crystalline anatase TiO_2_ hydrosol obtained by an aging process at room temperature. The experimental results highlighted that the use of sodium alginate as a binding agent is effective in favoring the distribution of TiO_2_ nanoparticles on paper surface, although some aggregations occurred, especially in paper sheets, with higher TiO_2_ loadings. Furthermore, the adhesion of the TiO_2_ nanoparticles to the cellulosic fibers was enhanced, as confirmed by the fact that photocatalytic papers can be used repeatedly while retaining their photocatalytic activity. Finally, the positive interactions between the abundant hydroxyl groups on the surface of TiO_2_ with the hydroxyl groups in both sodium alginate and cellulose allow to enhance the mechanical properties of the synthesized photocatalytic paper in terms of better tensile index, breaking length, tear and burst index, and air permittivity.

## Figures and Tables

**Table 1:** Crystallite size of TiO_2_ nanoparticles calculated by Debye–Scherrer method.

2θ	FWHM	Crystallite Size (nm)
25.23°	1.08	7.88
37.71°	1.12	7.89
47.72°	1.21	7.50
62.54°	1.89	5.14

**Table 2:** Rate constant K for the degradation of methylene blue in water with different TiO_2_-modified paper sheets and TiO_2_ nanoparticles at 365 nm UV light irradiation.

Catalyst	K(min^−1^) × 10^−3^ ± Error Limit × 10^−4^	Coefficient of Determination, R^2^
TiO_2_@365nm	21.7 ± 6.0	0.996
Paper-1.0TiO_2_	0.82 ± 0.2	0.996
Paper-0.5TiO_2_	0.77 ± 0.4	0.982
Paper-0.250 TiO_2_	0.41 ± 0.3	0.971
Paper-0.125TiO_2_	0.34 ± 0.4	0.931
